# Primary extra-nodal non-Hodgkin lymphoma in buttock soft tissue: A rare case report

**DOI:** 10.1097/MD.0000000000013550

**Published:** 2018-12-10

**Authors:** Xiang Meng Li, Hai Song Zhang, Xiao Li Dai, Jian Hong Shi

**Affiliations:** a Hematology Department of the Affiliated Hospital of Hebei University; b Head of the Central Laboratory of Hebei University, Hebei province, China.

**Keywords:** buttock soft tissue, extra-nodal lymphoma, non-Hodgkin lymphoma, PET/CT

## Abstract

**Rationale:**

Primary extra-nodal non-Hodgkin lymphoma (PE-NHL) arising in the region of the buttocks is rare. After reviewing the literature from the last 20 years, we found only 3 reported lymphomas originating from soft tissue of the buttocks. In our case, positron emission tomography/computed tomography (PET/CT) was performed for the first time, both before and after treatment, to determine the initial stage of PE-NHL and the curative effects of treatment.

**Patient concerns:**

We report the case of a 71-year-old woman who was admitted to our hospital due to pain, skin redness, rising skin temperature, and swelling in the right hip.

**Diagnoses:**

After an initial misdiagnosis of local infection, a histological examination and PET/CT were performed which revealed evidence of non-Hodgkin marginal zone B cell lymphoma of Ann Arbor stage II.

**Interventions:**

Following unsuccessful treatment with cephalosporin, the patient was successfully treated with rituximab combined with cyclophosphamide, doxorubicin, vincristine, and prednisone (R-CHOP) chemotherapy.

**Outcomes:**

Comparison of the PET/CT scans taken before and after treatment showed that the lesion size had decreased, as had the fluorodeoxyglucose (FDG) uptake seen in the subcutaneous tissue of the right buttock with standardized uptake value max (SUVmax) 11.6 versus 2.5, respectively. Subsequently, no relapse or distant metastasis has been detected.

**Lessons:**

Young doctors should suspect PE-NHL in similar cases. PET/CT is valuable in the diagnosis and treatment of PE-NHL, as well as for accurately determining PE-NHL stage and aggressiveness.

## 1 Introduction

Non-Hodgkin lymphoma (NHL) is associated with proliferation and differentiation of various immune cells in the immune response of lymphoid tissues. This type of malignant tumor of the immune system usually grows in the form of solid tumors in abundant lymphoid tissues and organs, including lymph nodes, tonsil, spleen, and bone marrow. These tumors can originate from any part of the body in addition to lymphoid tissues.^[1]^ Primary extra-nodal (PE)-NHL is defined as the first occurrence of NHL in any extra-nodal tissues or organs whose main lesions originate from lymph nodes. The most commonly involved extra-nodal organs are the gastrointestinal tract, Waldeyer ring, and nasal cavity, while primary extra-nodal non-Hodgkin lymphoma (PE-NHL) arising from the spleen, soft tissue, mediastinum, and other tissues is rare. PE-NHL arising in buttock soft tissue is extremely rare. Only a few case reports are available in the literature. After reviewing the literature from the last 20 years, we found only 3 reported cases of lymphoma originating from soft tissue of the buttocks.^[2–4]^ Yang Jing^[5]^ reviewed 3724 cases of lymphoma treated in the First Affiliated Hospital of Sun Yat-sen University from 1999 to 2010. Seven cases of primary soft tissue lymphoma were found. Among these, 3 cases were found in lower extremities, 2 cases in the psoas muscle, 1 case in the back, and 1 case in the sternocleidomastoid muscle.^[5]^ Therefore, this type of lymphoma is likely to be misdiagnosed.

## 2 Case report

A 71-year-old woman was admitted to our hematology department complaining of swelling with pain, skin redness, and increasing skin temperature in the right hip. There was no history of physical or psychological diseases, alcohol abuse, or familial history of similar diseases. Physical examination showed a red mass in the right hip and a long strip of mass in the right groin area; however, no superficial lymph nodes were found. Moreover, there was no enlargement of the liver or spleen. The patient was initially diagnosed with local infection and prescribed cephalosporin which had no obvious beneficial effects.

**Figure 1:**
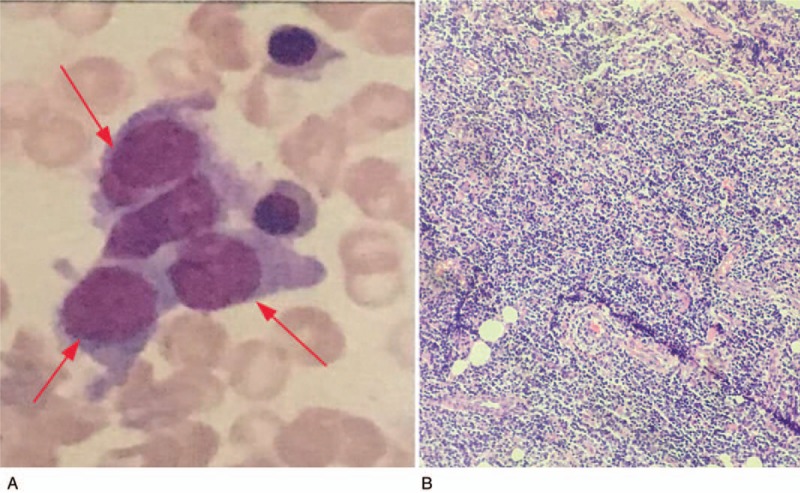
A: Bone marrow imaging of abnormal lymphocytes reveals irregularly shaped cell nucleus. Magnification, × 400. 1B: Right groin area lymph node biopsy pathology showing germinal center and lymphoid structure distinct from normal lymphoid tissue. Magnification, × 200.

**Figure 2:**
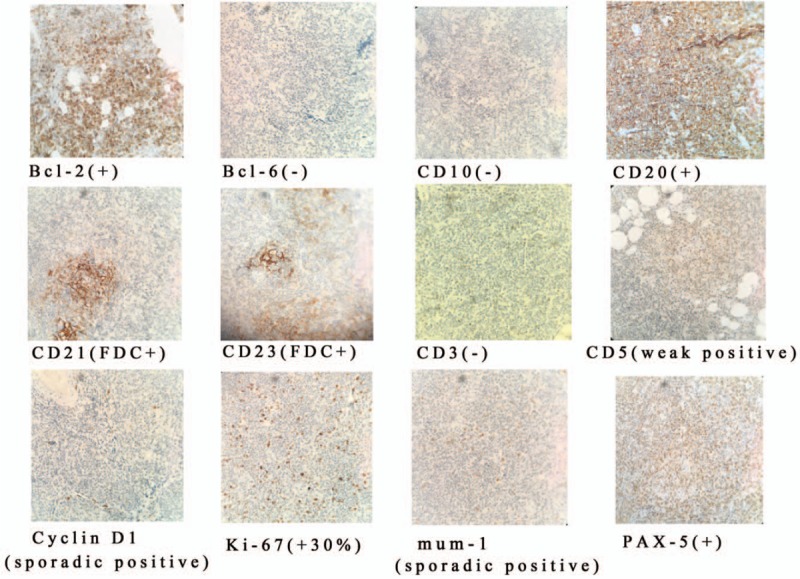
Immunohistochemical results of right groin area lymph node biopsy.

**Figure 3:**
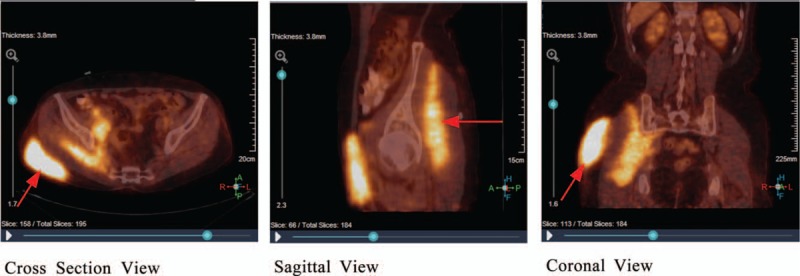
PET/CT scan of the tumor before treatment. PET/CT = positron emission tomography/computed tomography.

**Figure 4:**
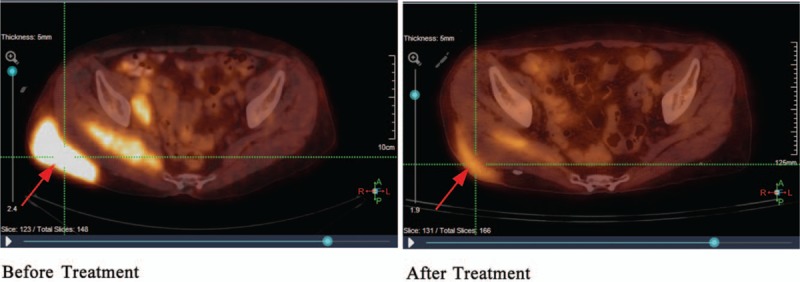
Comparison of the PET/CT results before and after treatment. PET/CT = positron emission tomography/computed tomography.

Subcutaneous puncture of the right buttock suggested that lymphoid tissue had hyperplastic lesions. Abnormal cells were found in the bone marrow, which suggested that lymphocyte bone marrow infiltration was considerable (Fig. 1 A). Right groin area lymph node biopsy pathology results showed that abnormal hyperplastic lymphoid tissue invasion was visible, the nucleus of the hyperplastic lymphoid cell was irregularly shaped, the germinal center was atrophied, and the lymphoid structure was distinct from normal lymphoid tissue (Fig. 1 B). The immunohistochemistry results of the right buttock tumor and right groin area lymph nodes were positive for CD5 (weak positive), CD20, CD21, CD23, and Bcl-2, but negative for CD3, CD10, and cyclin D1 (Table 1, Fig. 2). These results suggest that the buttock tumor and abnormal lymph nodes were both non-Hodgkin marginal zone B cell lymphomas.^[6,7]^ positron emission tomography/computed tomography (PET/CT) scans showed increased non-uniform fluorodeoxyglucose (FDG) uptake, with standardized uptake values (SUV)max of 11.6 in the subcutaneous tissue of the right buttock, SUVmax of 9.1 in the right gluteal muscle space, and SUVmax of 9.9 in multiple nodules on the right pelvic wall and right inguinal region (Fig. 3). With respect to tumor aggressiveness in the same side of the diaphragm, the disease was considered to be PE-NHL of Ann Arbor stage II.^[8]^ The patient was subsequently treated with 4 courses of rituximab combined with cyclophosphamide, doxorubicin, vincristine, and prednisone (R-CHOP) chemotherapy. Comparison of the PET/CT scans before and after treatment showed that the lesion had decreased, as had FDG-uptake in the subcutaneous tissue of the right buttock, with SUVmax of 11.6 vs 2.5, respectively (Fig. 4).

**Table 1:**
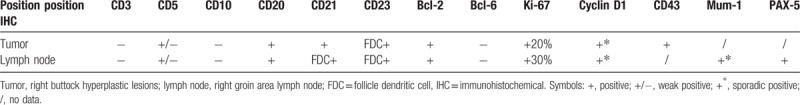
The comparison of immunohistochemical results of tumor and right groin area lymph nodes.

The patient was followed up for 3 months. The hip lesions had significantly narrowed with skin color deepening, and the pain was gradually relieved. Subsequently, no relapse or distant metastasis has been detected.

The patient provided informed consent. The study design was approved by the appropriate ethics review board and was CARE compliant.

## 3 Discussion

NHL is disseminated non-randomly and predictively through adjacent lymphatic channels and other lymphatic structures. The manifestations of lymph node involvement depend primarily on the location of the primary lesion and the local lymphatic drainage vessel.^[9]^ There are 2 channels of lymphatic drainage in the buttocks that allow lymphoid tissue from gluteal lymph nodes to flow into inguinal nodes and, eventually, into the lumbar trunk.^[10]^

Due to lack of both first-hand knowledge and reports in the literature, symptoms of swelling with pain, skin redness, and rising skin temperature are commonly considered typical infection symptoms in the early stages of treatment. Moreover, patients are often initially misdiagnosed as having a local infection and treated with cephalosporins (as was the case with our patient) and other anti-infective drugs, but without obvious effect. We found reports of 3 cases similar to ours through reviewing the literature of the previous 20 years.^[2–4]^ Scally et al^[2]^ reported the case of a 51-year-old woman with pain in her waist and left hip for 3 weeks who was misdiagnosed with sciatica, despite the fact that a lumbar spinal nerve root sheath angiogram showed no abnormalities. Subsequently, a mass appeared on her left hip as the pain gradually worsened; CT showed increased density of the buttock mass, and she was finally diagnosed as having B-cell NHL by buttock mass biopsy. Utkan et al^[3]^ reported the case of a 68-year-old man with a history of swelling and pain in his right buttock for 2 months. Histopathological examination of his inguinal lymph nodes and hip mass suggested tuberous sclerosis Hodgkin disease. Katsura et al^[4]^ treated a 52-year-old woman with left hip pain for 3 weeks. Her laboratory tests showed no abnormalities; however, enhanced CT revealed an early stage mass in her left gluteus medius muscle. Finally, her buttock mass was diagnosed as diffuse large B cell lymphoma (DLBCL) by biopsy.

An initial diagnosis of non-canonical PE-NHL may not be made due to lack of effective examination standards. Definitive diagnosis of PE-NHL requires the biopsy of lymph nodes, which is considered the gold standard for diagnosing NHL. Recently, the National Comprehensive Cancer Network (NCCN) guidelines recommended PET/CT for primary staging, early or final response evaluation, and determining the prognosis of lymphoma.^[11]^ Some reports have revealed that the sensitivity and specificity of PET/CT for extra-nodal lymphoma were 88% to 97% and 100%, respectively.^[12,13]^ Therefore, PET/CT may be the most suitable option for the diagnosis of patients who are highly suspected of having PE-NHL but reject invasive examination. PET/CT is vital for improving staging accuracy, and can ensure that patients are neither under- or overtreated.^[14]^ At the same time, PET/CT is one of the best methods for diagnosing the initial stages of PE-NHL and for re-staging after treatment.^[15]^ We concluded that, while destructive tumors originating in buttock soft tissue are more common in soft tissue sarcoma, PE-NHL should be considered in the differential diagnosis. A timely, accurate diagnosis of PE-NHL can clarify the course of treatment and improve the prognosis of these patients.

## Acknowledgments

Professional English language editing was provided by Editage.

## Author contributions

**Project administration:** Xiao Li Dai.

**Writing – original draft:** Xiang Meng Li.

**Writing – review & editing:** Hai Song Zhang, Jian Hong Shi.
